# Study on Stability and Stability Mechanism of Styrene-Acrylic Emulsion Prepared Using Nanocellulose Modified with Long-Chain Fatty Acids

**DOI:** 10.3390/polym11071131

**Published:** 2019-07-03

**Authors:** Heng Zhang, Hongyan Yang, Junliang Lu, Jinyan Lang, Hongkun Gao

**Affiliations:** 1 College of Marine Science and Biological Engineering, Qingdao University of Science & Technology, Qingdao 266042, Shandong, China; 2 Key Laboratory of Biomass Chemical Engineering of Ministry of Education, Zhejiang University, Hangzhou 310027, China

**Keywords:** modified nanocrystalline cellulose, long-chain fatty acids, hydrophilic lipophilicity, styrene-acrylic emulsion, stability mechanism

## Abstract

In this study, nanocrystalline cellulose (NCC) was grafted with lauric acid, palmitic acid, and stearic acid and used as stabilizer to prepare styrene butyl acrylate emulsion. The properties of the emulsion were determined, and the mechanism of modified NCC (MNCC) stabilized emulsion was analyzed. Results showed that long-chain fatty acids were grafted to NCC through esterification initiated at a low temperature. When the dosage of L-MNCC, P-MNCC, and S-MNCC was 0.05%, the styrene-acrylic emulsion had 92.5%, 94.2%, and 96.3% conversion rates, respectively, and exhibited good dilution, pH, Ca^2+^, and centrifugal stability. The particle size of styrene-acrylic emulsion was approximately 460 nm, and the absolute value of the Zeta potential increased with the MNCC concentration. According to the images of optical microscopy and the transmission electron microscope, the MNCC was adsorbed onto the surface of styrene-acrylic emulsion droplets. The synergistic effect from the electrostatic repulsion of MNCC, the hydrophile lipophilicity of MNCC, and the spatial hindrance of the MNCC adsorption layer provided good stability for the styrene-acrylic emulsion. Therefore, MNCC could replace traditional surfactants in stabilizing emulsion.

## 1. Introduction

Emulsion with a large oil/water interface is a thermodynamically unstable system [1]. Adding surfactants could effectively reduce the surface tension and promote the long-term stability of the emulsion [2,3]. Stabilized emulsions could also be achieved using highly dispersible solid powders, such as carbon black, silica, clay, and calcium carbonate [4]. In the past 10 years, the increasingly widespread application of emulsion in food and biomedicine has required stable particles with better biocompatibility, biodegradability, and sustainability [5]. Therefore, research on emulsion stability focuses on transforming traditional surfactants to biogenic derivatives, such as cellulose, hemicellulose, lignin, chitin, starch, protein, and their derivatives [6,7]. Cellulose derived from wood or cotton has attracted considerable attention in recent years due to its biodegradable, biocompatible, and renewable character [8,9]. Chmielarz [9] prepared brush copolymers of cellulose at low catalyst concentration by the ATRP method. It is expected that those polymer brushes may find application as pH-and thermo-sensitive drug delivery systems. Nanocrystalline cellulose (NCC) has excellent properties, such as high purity, DP, crystallinity, and hydrophilicity [10]. Winuprasith [11] et al. reported that the microcrystalline cellulose (MFC) extracted from mangosteen peel stabilized the emulsion without the help of surfactants, and this effect increased with the decrease in fiber size and the increase in MFC concentration. Laitinen [12] et al. also found that NCC exhibited excellent stability in emulsion.

Hydrophobically modified NCC could stabilize emulsions because its hydrophilic lipophilic property allows good adsorption on oil-water surfaces [13]. Stark [14] et al. chemically modified NCC using rapeseed oil fatty acid methyl ester through transesterification to obtain a modified NCC that had certain hydrophobicity and could be combined with polylactic acid in nano-materials with good plasticizing effect. Cunha [15] et al. surface modified NCC with chemically modified lauryl chloride to endow the NCC with a hydrophobic property and decreased hydrophilicity. They also proved that the modified NCC could stabilize the oil in water emulsion without surfactants. Many researchers also provided the mechanism for hydrophobically modified NCC stabilized emulsion. On the one hand, Hydrophobic groups of hydrophobic modified nanofibers extend into the interior of microemulsions, and hydrophobic groups associate with each other to induce thickening. Additionally, the elastic 3D network structure formation, which effectively inhibits emulsion flocculation [16,17,18]. On the other hand, the hydrophilic part of the nanocellulose chain greatly increased its solubility in water and enhances its dispersion in aqueous solution [16]. Therefore, the hydrophobic-modified NCC could be used as a stabilizer and emulsifier and had incomparable advantages over small molecular surfactants. The application of modified NCC instead of traditional surfactants in emulsion stabilization could expand the application scope of biomass resources, reduce the dependence on petrochemical products, and contribute to the protection of the environment [9,19].

In this study, NCC was prepared through sulfuric acid hydrolysis, and long-chain fatty acids were used to initiate NCC graft modification with FeSO_4_/H_2_O_2_ as an initiator. Styrene-acrylic emulsion was prepared using modified NCC (MNCC) instead of the traditional surfactant. The stability and stability mechanism of styrene-acrylic emulsion were analyzed by testing the solid content, conversion rate, physicochemical stability, particle size, and zeta potential as well as conducting SEM characterization.

## 2. Materials and Methods

### 2.1. Materials

Ammonium ceric sulfate and FeSO_4_ were purchased from China Shanghai Pierre Chemical Reagent Co., Ltd, Shanghai, China. H_2_O_2_ (30%) and ammonium persulfate were obtained from Tianjin Dingshengxin Chemical Co., Ltd, Tianjin, China. Concentrated sulfuric acid (98%) was purchased from Yantai Sanhe Chemical Reagent Co., Ltd, Yantai, China. Tianjin BASF Chemical Co., Ltd. (Tianjin, China) provided the lauric acid, palmitic acid, and stearic acid. Butyl acrylate and calcium chloride were obtained from Tianjin Bodi Chemical Co., Ltd, Tianjin, China. Chemical Reagent Co., Ltd. (Shanghai, China) provided the anhydrous ethanol, styrene, sodium hydroxide, and ammonia. All these chemical reagents were of an analytical grade. Medicinal grade microcrystalline cellulose was obtained from Chengdu Kelon Chemical Reagent Factory, Tianjin, China. The dialysis bag MD3500 was acquired from Viskase Company, USA (Lombard, IL, USA).

### 2.2. Experimental Method

#### 2.2.1. Preparation of NCC

MFC was added to a one-neck flask (250 mL) and heated at 45 °C in a water bath. The prepared H_2_SO_4_ (60%) solution was slowly added, and the solution was mixed well by a medium-sized propeller agitator (material: PTFE). A milky white NCC suspension was obtained after 2.5 h. The heating was stopped, and cooling to room temperature followed in half an hour. The reaction was terminated by adding 500 mL deionized water. The obtained NCC suspension was then centrifuged for 5 min at a speed of 10,000 r/min, and the acid solution in the upper layer was removed, which was repeated 2 to 3 times. Centrifugation was continued until an NCC colloid appeared in the upper layer, which was deposited into the treated dialysis bag and subjected to dialysis to achieve neutral pH. Lastly, a blue-light NCC colloidal solution was obtained, which did not stratify for three months.

#### 2.2.2. Preparation of MNCC

Fifty mL ethanol was used to dissolve lauric acid 0.50 g (palmitic acid 0.64 g, stearic acid 0.71 g). Additionally, an appropriate amount of NCC suspension were mixed with fatty acid ethanol solution by stirring with a speed of 180 r/min. The stirring temperature was increased to 50 °C in half an hour. Additionally, 26 μL H_2_O_2_ (30%), 0.0070 g FeSO_4_, and 1–2 mL 0.5 mol/L H_2_SO_4_ solution were added successively. The grafting reaction was initiated, and the reaction lasted for 8 h with stirring under 50 °C. The mixed suspension containing grafted NCC was then obtained. The suspension was filtered by a Vacuum filter bottle and washed repeatedly with water and ethanol. Then the upper filter cake was collected with tweezers and placed into the Rapid Glass Dryer (containing discolored silica gel) to wait for the ethanol solvent to evaporate naturally. After 2 hours, the MNCC was obtained (lauric acid modified nanocrystalline cellulose, L-MNCC, palmitic acid modified nanocrystalline cellulose, P-MNCC, and stearic acid modified nanocrystalline cellulose, S-MNCC).

The NCC was modified with different fatty acids as follows: with lauric acid under the reaction conditions of 0.8% FeSO_4_/H_2_O_2_ (molar ratio 1:1) as an initiator and reacted at 50 °C for 8 h, with palmitic acid under the reaction conditions of 1.2% FeSO_4_/H_2_O_2_ (molar ratio 1:1) used as an initiator and reacted at 55 °C for 20 h, and with stearic acid under the reaction conditions of 1.2% FeSO_4_/H_2_O_2_ (molar ratio 1:1) as an initiator and reacted at 60 °C for 24 h.

#### 2.2.3. Preparation of MNCC Suspension

Then, 0.1 g MNCC powder was added into 100 mL deionized water and disposed into 1 g/L MNCC suspension, which was dispersed by ultrasound for 30 min and stored in bottles for reserve.

#### 2.2.4. Preparation of Styrene-Acrylic Emulsion

As shown in Table 1, a certain amount of MNCC suspension was dispersed by an ultrasonic disperser for 30 min and added to the flasks, and the temperature was increased to 75 °C. A small amount of ammonium persulfate initiator was added, and the temperature was increased to 86 °C. The monomer mixture was made of styrene and butyl acrylate was dripped with a constant pressure funnel for 4 h, and ammonium persulfate initiator was simultaneously incorporated. A small amount of initiator was added during heat preservation for 1 h and then cooled to room temperature. Lastly, the styrene-acrylic polymer emulsion was obtained through filtration.

### 2.3. Reaction Principle

**Figure 1:**
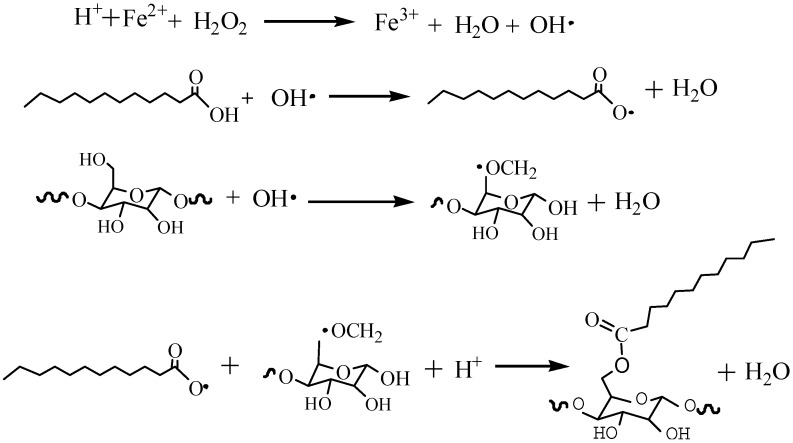
Reaction principle of synthetic MNCC.

The reaction process of esterification and grafting modification of nanocellulose with fatty acids is shown in Figure 1. Hydroxyl radicals can be produced by H_2_O_2_ and FeSO_4_ in the reaction system. Hydroxyl radicals can capture hydrogen atoms on fatty acids and NCC to form water, fatty acid radicals, and NCC radicals, respectively [20,21,22]. Fatty acid radicals and NCC radicals can be esterified to obtain MNCC [22].

### 2.4. Methods

The NCC colloidal suspension was dried and powdered, and small amounts of NCC and MNCC solid powders were obtained. The infrared spectrum was then determined through the KBr pressing method with a resolution of 4 cm^−1^ in the region of 4000 cm^−1^ to 500 cm^−1^ by using Fourier infrared spectrometer (VECTOR22). The powders were then analyzed by X-ray diffraction (XRD) tester (DX-2700) at room temperature with a scanning rate of 0.02°/min and a diffraction angle of 2θ within a 10° to 60° range.

Small amounts of NCC suspension, MNCC suspension, and styrene-acrylic emulsion were separately diluted in distilled water at a ratio of 1:10 until a transparent solution was obtained. The particle size distribution and potential of latex particles were measured by the Malvern ZetaSizer Nano-ZS90 laser particle size analyzer (Shanghai, China).

Exactly 1.5000 g of the prepared emulsion was placed in a weighted drying bottle and, subsequently, inside an oven and dried at 100 °C until a constant weight was achieved. Solid content was then calculated, followed by the conversion rate, according to the theoretical solid content.

The emulsion was diluted with different multiple times, stirred evenly, and sealed for 48 h. In a beaker, 20 mL of emulsion and different pH acid-alkali (H_2_SO_4_, NaOH, and ammonia) solutions were added, and the mixture was allowed to stand for 48 h. A 0.5% Ca^2+^ solution was mixed with emulsion in a 4:1 ratio and sealed for 48 h. Then, 20 mL of emulsion was centrifuged at speeds of 1000, 2000, 3000, 4000, and 5000 r/min for 10 min. Lastly, the occurrence of delamination and flocculation in the emulsion was observed.

The emulsion was centrifuged at 4000× *g* r/min for 10 min and then placed in the separation funnel. The lower layer of emulsion was released, and the volume of the upper layer (oil) was measured. The proportion of stable emulsion was calculated.

The microscopic morphology of emulsion microspheres was observed using the optical microscope (PM6000, Nanjing Jiangnan Yongxin Optical Co., Ltd., Nanjing, China) and the transmission electron microscope (JSM-2100Plus, JEOL, Tokyo, Japan).

## 3. Results and Discussion

### 3.1. Preparation and Analysis of MNCC

#### 3.1.1. Infrared Characterization of MNCC

**Figure 2:**
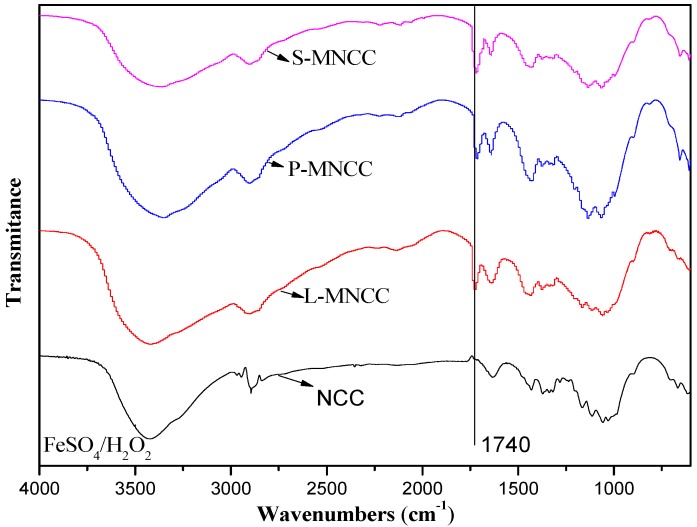
IR spectra of MNCC.

As shown in Figure 2, the stretching vibration peak of the –CH_2_– long-chain alkyl appeared at 2918 and 2850 cm^−1^ in the infrared spectrum of MNCC, which indicates that long-chain fatty acids had been grafted on the NCC. Moreover, the vibration peak of saturated ester bond appeared at 1740 cm^−1^, and the characteristic peak of –COO– appeared at 1582 cm^−1^. This finding indicates that the hydroxyl of NCC reacted with the acid group of long-chain fatty acids to produce ester groups, and the nanocellulose and long-chain fatty acids successfully underwent esterification copolymerization [22]. Hence, long-chain fatty acids could be grafted to NCC under certain reaction conditions by using FeSO_4_/H_2_O_2_ system as an initiator to obtain stable MNCC products.

#### 3.1.2. XRD Analysis of MNCC

**Figure 3:**
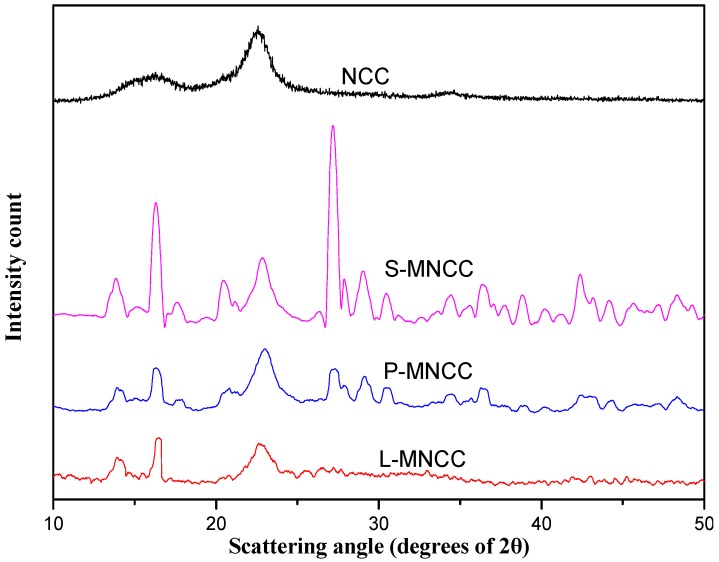
XRD patterns of MNCC.

Figure 3 provides a comparison of the XRD images of NCC with those of L-MNCC, P-MMCC, and S-MNCC, which was prepared by using FeSO_4_/H_2_O_2_ system as an initiator. The L-MNCC, P-MNCC, and S-MNCC exhibited diffraction peaks at I_002_ (2θ = 22.58°), I_am_ (2θ = 16.38°), and I_004_ (2θ = 34.54°), respectively, which indicated that the FeSO_4_/H_2_O_2_ system successfully initiated the NCC grafting reaction. Furthermore, the basic crystal structure of the NCC was also maintained [23]. The XRD image of the L-MNCC was substantially similar to that of NCC, and the crystal structure did not remarkably change. The XRD images of P-MNCC and S-MNCC showed diffraction peaks at other positions and changed the crystal structure. This phenomenon occurred because the palmitic acid and stearic acid have a longer carbon chain structure than lauric acid. The steric hindrance was large, and the crystal structure of NCC was slightly changed after the grafting at NCC [20,23].

#### 3.1.3. Particle Size Test of MNCC

**Figure 4:**
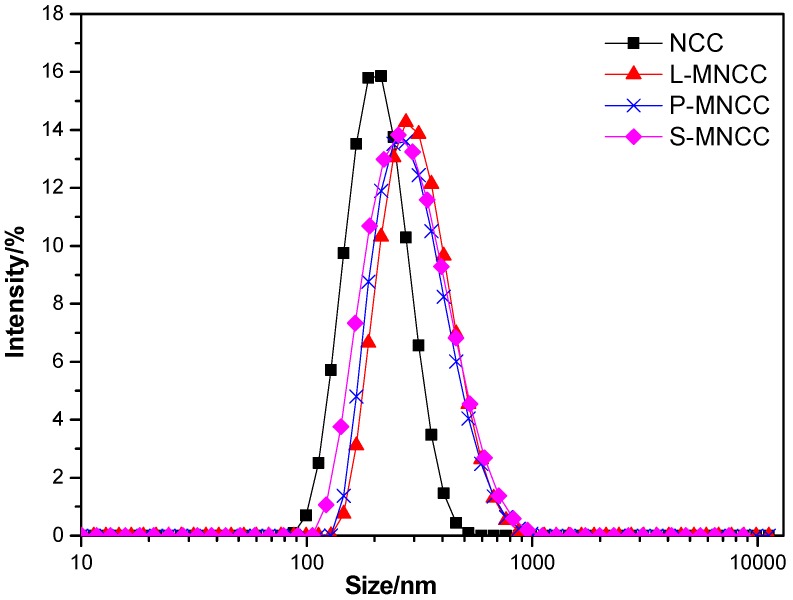
Particle size distribution of MNCC.

Figure 4 showed that the average particle size of MNCC, which was modified by fatty acids, was slightly increased than NCC. The average particle size, the particle size distribution coefficient, and the specific surface area were 168 nm, 0.255, and 19,260 m^2^/kg, respectively, for NCC; 255 nm, 0.297, and 17,500 m^2^/kg, respectively, for L-MNCC; 244 nm, 0.308, and 17,400 m^2^/kg, respectively, for P-MNCC; and 247 nm, 0.319, and 17,430 m^2^/kg for S-MNCC. The difference of particle size, the particle size distribution coefficient, and the specific surface area of L-MNCC, P-MNCC, and S-MNCC were very small. The particle size of MNCC were slightly different from that of NCC, likely because the molecular weight of MNCC increased slightly after the hydrophobic group was grafted, so the particle size of MNCC increased slightly. Although the particle size of MNCC was slightly increased, the particle size was still in a nanometer range. Moreover, the distribution coefficient of the particle size of MNCC was larger than that of NCC, which indicates that the particle size distribution of MNCC was wide, and a certain degree of flocculation occurred during the reaction of fatty-acid-grafted NCC. The particles of MNCC were enlarged, and the specific surface area of MNCC was reduced relative to that of NCC. After being grafted with long-chain fatty acids, NCC reactivity was reduced to some extent. In summary, the average particle size of grafted NCC was 244 to 255 nm, which was not a large increase relative to that of NCC. The particle size remained at the nanometer scale and had a large specific surface area.

### 3.2. Emulsification and Stability Analysis of Styrene-Acrylic Emulsion

#### 3.2.1. Effect of MNCC Dosage on the Solid Content and Conversion of Emulsion

In emulsion polymerization, styrene and butyl acrylate were the reaction monomers, and ammonium persulfate was the initiator. Table 1 (from Section 2.2.4) showed the main compositions and indicated that the theoretical solid content of styrene-acrylic emulsion was 40%.

Certain amounts of L-MNCC, P-MNCC, and S-MNCC were weighed and added to deionized water and dispersed by ultrasound for 30 min. Styrene-acrylic emulsion polymerization was then conducted, according to the proportion shown in Table 1, and the solid content and conversion rate of the emulsion obtained were calculated. The test results are shown in the following figure.

**Figure 5:**
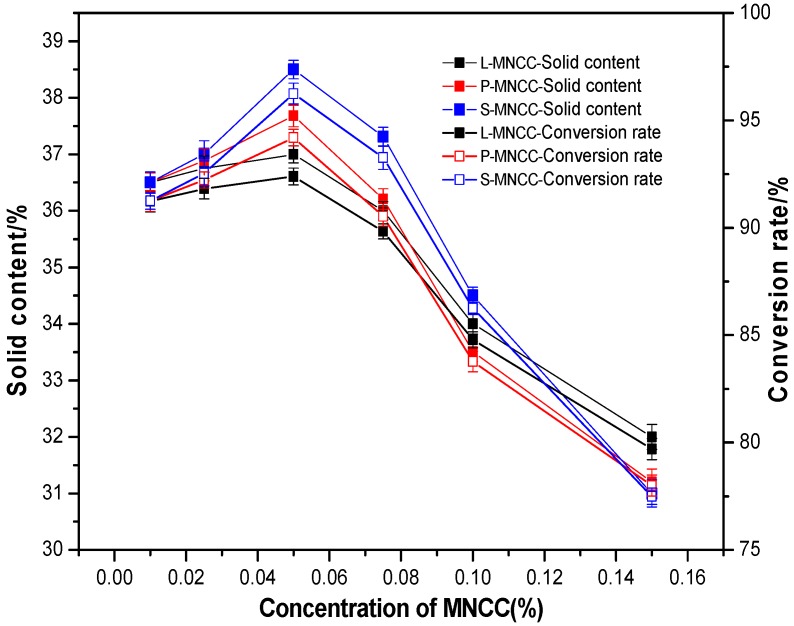
Effect of MNCC dosage on the solid content and conversion of styrene-acrylic emulsion.

Figure 5 showed that, when MNCC dosage was less than 0.05%, the emulsification of MNCC was weaker. With the increase of MNCC dosage, the emulsifying effect was enhanced, a large number of micelles were generated, and the nuclear growth process accelerated, which made the emulsion solid content and conversion rate increase. When the dosage of MNCC was 0.05%, the solid contents of styrene-acrylic emulsion were 37%, 37.68%, and 37.68%, and the conversion rates were 92.5%, 94.2%, and 96.25%, respectively. When the dosage of MNCC was higher than 0.05%, the solid content and conversion rate of styrene-acrylic emulsion showed a decreasing trend with the increasing MNCC dosage. It might because the viscosity of styrene-acrylic emulsion increased significantly as a result of the thickening effect of MNCC, which resulted in the contact between monomers being less than when the little dosage of MNCC was used. Higher solids and conversion rates were observed for emulsions containing P-MNCC and S-MNCC compared with L-MNCC, because the carbon chains of the hydrophobic groups of P-MNCC and S-MNCC were longer than those of L-MNCC. They had better hydrophobic ability, which could better form micelles and help with emulsion polymerization [24]. This finding indicated that the length of the hydrophobic carbon chain had a certain effect on the conversion rate of the emulsion. The longer the hydrophobic carbon chain was, the better the emulsification, and the higher the conversion rate of styrene-acrylic emulsion that would be obtained.

#### 3.2.2. Effect of MNCC Dosage on Emulsion Particle Size

A certain amount of styrene-acrylic emulsion was diluted for 10 times. Then, the particle size was tested. The test results are shown in the following figure.

**Figure 6:**
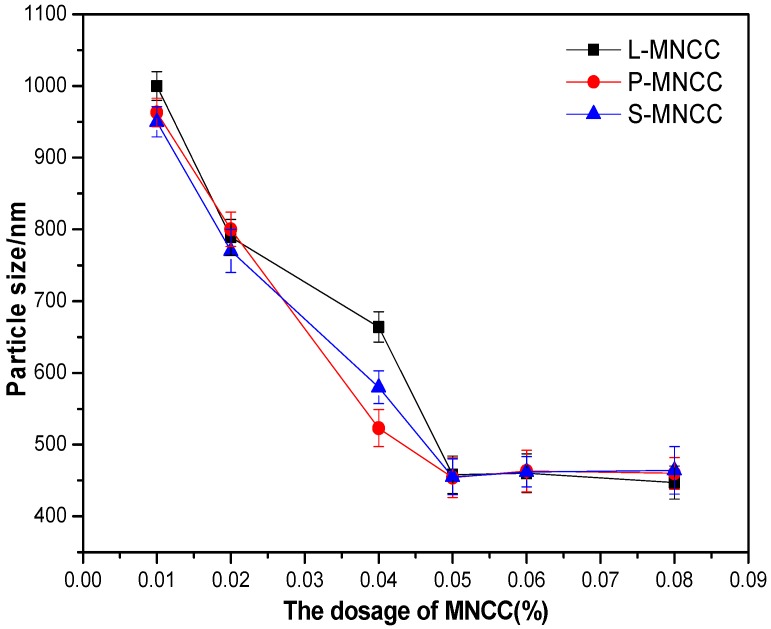
Effect of MNCC dosage on the particle size of emulsion.

Figure 6 showed that, when the dosage of MNCC was less than 0.05%, the particle size of the emulsion gradually decreased with the increase of the dosage of MNCC. When the dosage of MNCC was 0.05%, the emulsion particle size was stable at approximately 460 nm. With the increase of the dosage of MNCC, the emulsion particle size did not change. When the concentration of MNCC was small, the dispersion and emulsifying ability of MNCC was weak, and the stable emulsion could not be formed. As a result, droplets tend to aggregate, and the size of the emulsion formed was larger. When the concentration of MNCC increased, its emulsifying and dispersing ability increased. Hence, the aggregation of emulsion decreased, and the particle size of the emulsion decreased. When the dosage of MNCC reached 0.05%, MNCC could play a good emulsifying function, which could form micelles better and form stable emulsion. When the dosage of MNCC was greater than 0.05%, the effect of MNCC concentration on emulsion micelles decreased. MNCC was only dispersed in the emulsion. Hence, the particle size of the emulsion no longer changed. The three modified products of L-MNCC, P-MNCC, and S-MNCC had a little effect on the particle size of emulsion. When the emulsion was stable, the particle size of the emulsion was approximately 460 nm. The difference among the three modified products was reflected in the carbon chain length and hydrophobicity of hydrophobic groups. The formation of emulsion droplets was encapsulated by MNCC droplets, and the structure of the three modified products was not very different. Therefore, the particle size difference between the three modified products as surfactants was almost absent. Moreover, when the MNCC dosage was 0.05%, the particle size distribution coefficients particle size distribution coefficient of the emulsion were 0.297, 0.319, and 0.308, respectively. A small particle size distribution coefficient value indicates uniform particle size distribution. Therefore, a narrow distribution indicates the uniform particle size of emulsion. In summary, when the dosage of L-MNCC, P-MNCC, and S-MNCC was 0.05%, the particle size of emulsion was the smallest at 460 nm.

#### 3.2.3. Effect of pH, Ca^2+^, and Dilution on the Stability of Styrene-Acrylic Emulsion

The styrene-acrylic emulsion containing 0.05% L-MNCC was obtained. In addition, pH dilution and centrifuge stability tests were conducted, according to Table 2. Ca^2+^ stability test was conducted on the styrene-acrylic emulsion of different amounts of L-MNCC. The test results are shown in Table 2. The centrifugal stability of styrene-acrylate emulsion with different dosages of MNCC was tested under 4000 r/min condition. The result is shown in Table 2.

Table 2 showed that, when the L-MNCC dosage was less than 0.05%, different degrees of delamination were observed following the addition of Ca^2+^ to the emulsion. When the L-MNCC dosage was higher than 0.05%, the emulsion was stable, and no oil phase was detached from the emulsion system. This finding indicates that L-MNCC at a low concentration did not completely cover the droplet, and the emulsification and dispersion were weak. Therefore, the formed emulsion was not stable. In the case of adding Ca^2+^ (salt solution), oil-water separation was easily generated, which results in demulsification. In Table 2B, the pH stability test was conducted using a styrene-acrylic emulsion. In the acidic and neutral environment, the emulsion showed stability. However, when the NaOH solution was added to the emulsion, it was flocculated and stratified. This is possible because there was a peeling reaction between NaOH and L-MNCC [25]. Moreover, the addition of OH^-^ might have destroyed the stability of the emulsion internal charge, which results in the instability of the emulsion to produce flocculation. Therefore, replacing NaOH with ammonia (ammonia could not cause NCC to initiate peeling reaction) retested the alkali stability and found that flocculation and delamination did not occur. Therefore, it could be explained that strong alkali solution could cause MNCC to occur peeling reaction and degrade, destroy the structure of MNCC, and make it no longer have emulsifying capacity, which results in micelle breaking and flocculating and delamination of emulsion. However, the emulsion was stable under weak bases, and the addition of aqueous ammonia to the emulsion did not cause the sedimentation of emulsion.

**Figure 7:**
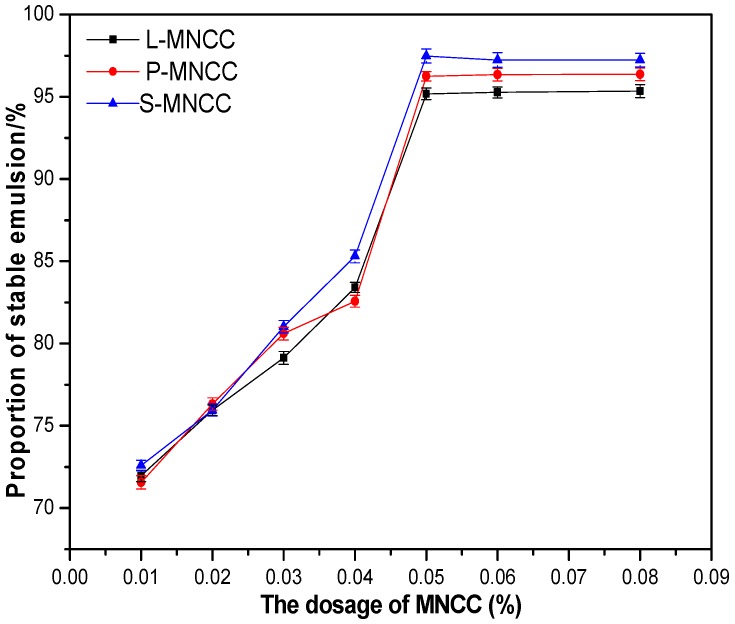
Effect of MNCC dosage on the centrifugal stability of emulsion.

Table 2C showed that the dilution of the solution did not destroy the stability of the emulsion. It showed that the dilution factor of the emulsion did not affect the stability of the emulsion. Table 2D showed that, under the action of high-speed centrifugation, the oil phase of the emulsion was slightly desorbed to form an oily liquid suspension layer. The emulsion exhibited a stable state when the centrifugal rotation speed was decreased. Figure 7 showed that, when the dosage of MNCC was less than 0.05%, the centrifugal stability of the emulsion continued to increase as the amount of MNCC increased. Moreover, the centrifugal stability of the emulsion was best when the amount of MNCC reached 0.05%. The hydrophobic properties of the three modified products were different. When the amount of MNCC was gradually increased, MNCC could cocoon the oil phase in the micelle. Hence, even if MNCC was subjected to the centrifugal force, most of the oil phase was difficult to detach. When the amount of MNCC exceeded 0.05%, the emulsion also exhibited better centrifugal stability. However, when the centrifugal speed reached 5000 r/min, instability occurred. The above results indicated that the emulsion had certain stability.

#### 3.2.4. Zeta Electric Potential Test of Styrene-Acrylic Emulsion

The blank styrene-acrylic emulsion containing no MNCC and the styrene-acrylic emulsion containing different contents of L-MNCC, P-MNCC, and S-MNCC were diluted for 1000 times into a transparent liquid. Then, the Zeta potential distribution of styrene-acrylic emulsion was tested with a Malvern laser particle size potential analyzer, and the test results are shown below.

**Figure 8:**
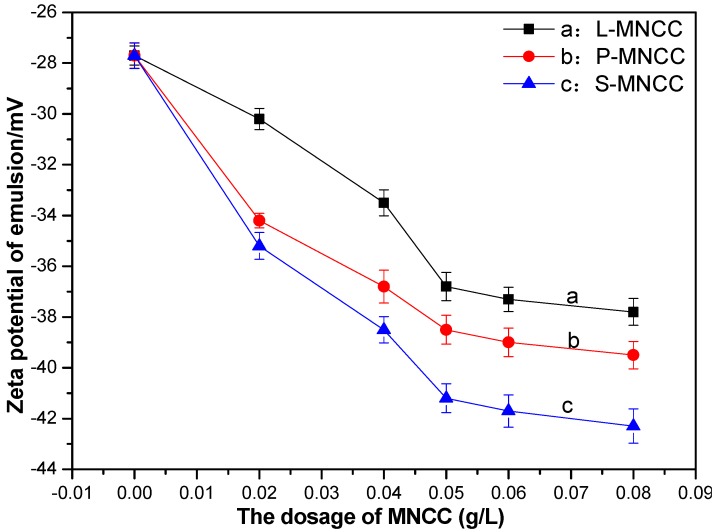
Effect of MNCC dosage on the zeta potential of styrene-acrylic emulsion.

Figure 8 showed that the zeta electric potential of the styrene-acrylic emulsion exhibited a negative electric potential. As the amount of MNCC increased, the absolute value of the Zeta electric potential of the styrene-acrylic emulsion increased, and the value of the zeta electric potential had a certain relation that, the greater the absolute value of the zeta electric potential had, the more stable the emulsion was with the stability of the emulsion [26]. According to the theory of colloidal stability, Van der Waals gravity made the droplets attract each other. When the droplets approached the surface of the electric double layer and overlapped, the electrostatic repulsion hindered further contact of the droplets [26]. The surface of the MNCC contains hydroxyl groups, according to the diffusion electric double layer theory. The MNCC was negatively charged in water. According to the styrene-acrylic emulsion test, when the amount of MNCC increased, the absolute value of the zeta electric potential of the styrene-acrylic emulsion increased. Therefore, the repulsion between the droplets increased, and the emulsion was more stable. When the amount of MNCC exceeded 0.05%, the absolute value of the zeta electric potential of the styrene-acrylic emulsion remained as increasing, but the increase was slow, because the excess MNCC could form micelles or dissociate in the emulsion, which can also increase the zeta electric potential of the styrene-acrylic emulsion system. By comparing the three curves of a, b, and c, with the increase of the carbon chain length of the hydrophobic group of MNCC, the zeta electric potential of the stabilized styrene–acrylic emulsion also had an increasing tendency, because the degree of substitution of L-MNCC, P-MNCC, and S-MNCC decreased. The hydroxyl content of the surface of the modified nanocellulose increased, so the zeta electric potential increased. This finding was consistent with the results of the previous tests on solid content and stability. As the carbon chain length of the MNCC hydrophobic group increased, the stability of the stabilized styrene–acrylic emulsion increased sequentially.

#### 3.2.5. Optical Microscopy Test of Styrene-Acrylic Emulsion

The styrene-acrylic emulsion containing 0.05% L-MNCC, P-MNCC, and S-MNCC was diluted 1000 times into a transparent liquid. Then, a small diluted emulsion was obtained and observed under an optical microscope. The test results were as follows.

**Figure 9:**
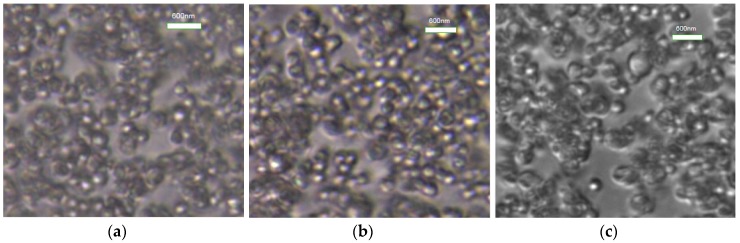
Optical microscopy of styrene-acrylic emulsion stabilized by MNCC. (**a**) L-MNCC, (**b**) P-MNCC, and (**c**) S-MNCC.

Figure 9 showed that the oil was dispersed into spherical droplets, and the NCC was coated on the surface of the droplets when the styrene-acrylic emulsion was magnified by 1600 times. Hence, MNCC could effectively coat the oil droplets to form a stable emulsion. The MNCC had a small particle size and was stable in the form of an emulsion. The optical microscope shows that the stability mechanism of the MNCC and the pickering emulsion were similar. In other words, the nano-sized solid particles adsorbed onto the surface of the emulsion monomer to stabilize the emulsion [11,27,28]. However, the MNCC also had a part of hydrophobic groups, which made the MNCC easier to stabilize oily droplets and enhanced the stabilizing effect. According to the principle of similar compatibility, the hydrophobic group of the MNCC clung to the inside of the oil droplet, and the hydroxyl group extended to the water phase. Hence, the MNCC was coated on the surface of the droplet to form an adsorption layer. Therefore, the droplets could be stably present in the aqueous phase.

#### 3.2.6. Transmission Electron Microscopy Analysis of the Emulsion

A blank styrene-acrylic emulsion (the preparation method was shown at Section 2.2.4, but no MNCC was added) and a styrene-acrylic emulsion containing different contents of L-MNCC, P-MNCC, and S-MNCC were diluted into a translucent liquid. A small amount of diluted emulsion was obtained and dropped on the copper net to let it dry naturally. The morphology of the styrene–acrylic emulsion droplets was then observed using a transmission electron microscope. The test results were as follows.

**Figure 10:**
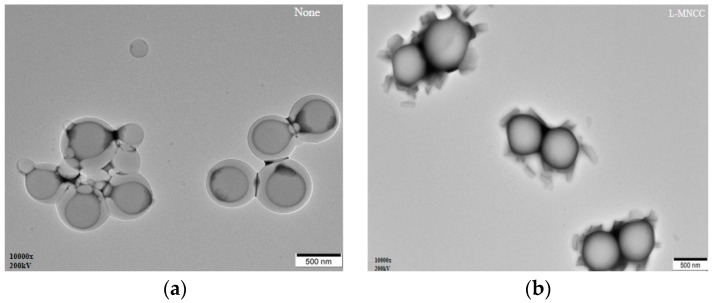

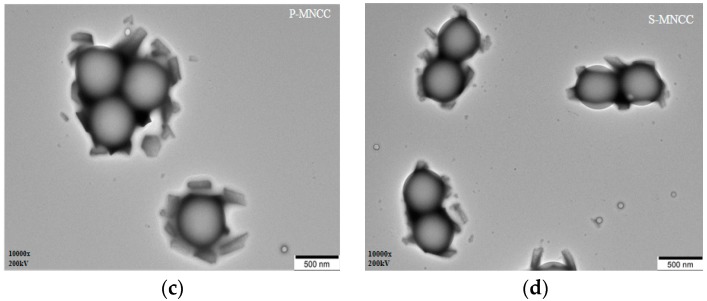
Transmission electron microscope image of styrene-acrylic emulsion: (**a**) None MNCC, (**b**) L-MNCC, (**c**) P-MNCC, and (**d**) S-MNCC.

Figure 10 showed that several small droplets were brought together to form a large agglomerate after the droplets of the styrene-acrylic emulsion were diluted in the absence of MNCC (Figure 10a). The degree of aggregation of the droplets was reduced, but the diameter of the individual droplets was increased to some extent after the addition of MNCC (Figure 10b–d). The MNCC coated around the droplets of styrene-acrylic emulsion could effectively stabilize and disperse the emulsion. Hence, the stability of the emulsion depended not only on the electrostatic repulsion of the MNCC but also on the hydrophilic and lipophilic structure of the MNCC. The hydrophobic group of the MNCC clung to the inside of the droplet, and the hydrophilic structure tended to the aqueous phase. Hence, it could be adsorbed on the surface of the droplet to stabilize the emulsion.

#### 3.2.7. Mechanism Analysis of MNCC-Stabilized Emulsion

The stability of the styrene-acrylic emulsion was mainly determined by The electrostatic exclusion of MNCC, the hydrophile lipophilic property of MNCC, and spatial hindrance of MNCC adsorption layer through the measurement of the particle size of the styrene–acrylic emulsion, the zeta potential distribution, the observation, and analysis by optical and transmission electron microscopy. The test results were as follows.

**Figure 11:**
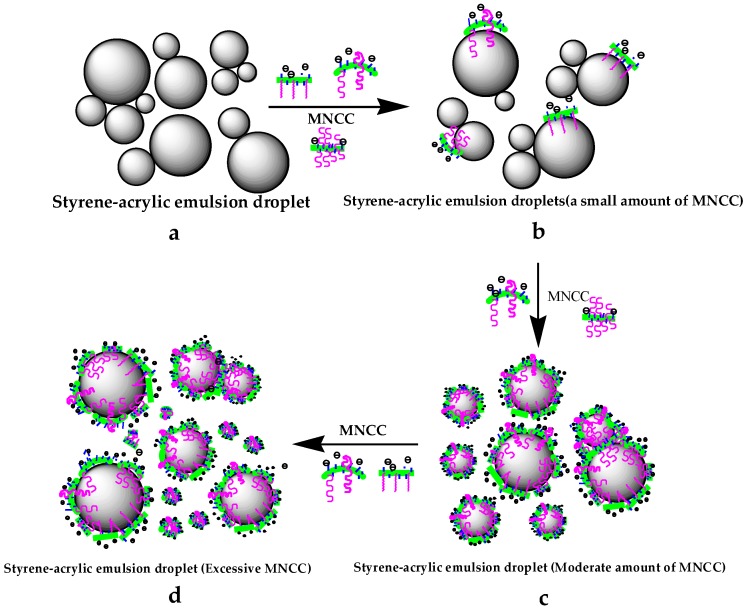
Mechanism of MNCC stabilizing styrene-acrylate emulsion.

Figure 11 showed that the particle size distribution of the styrene-acrylic emulsion droplets was not uniform, and the emulsion droplets of different sizes and sizes were easily aggregated when MNCC was not added (Figure 11a). The styrene-acrylic emulsion formed was unstable and was easily affected by external influences causing coalescence, instability, or flocculation at this time. Considering that MNCC had a certain hydrophilic and lipophilic ability, the hydrophilic group of the MNCC would cling to water, and the lipophilic group composed of the hydrophobic hydrocarbon long chain would extend to the inside of the droplet. Thereby, MNCC was adsorbed on the surface of the styrene–acrylic emulsion droplets (Figure 11b). However, the coating of the styrene-acrylic emulsion droplets could not be formed when the content of MNCC was small. Therefore, the stability was still not good, even though the stability of the styrene-acrylic emulsion at this time was improved to some extent. The MNCC could completely form the droplets of the styrene-acrylic emulsion when the concentration of MNCC reached a certain amount (Figure 11c). The contact between the droplets of the styrene-acrylic emulsion was greatly reduced, so the formed emulsion was further stabilized. Due to the hydroxyl structure on the surface of the MNCC, the surface of the MNCC exhibited negative electrons. Hence, the droplets and droplets were less likely to contact and aggregate due to electrostatic repulsion. Styrene-acrylic emulsions exhibited high stability. The excess MNCC existed in the aqueous phase in the form of micelles when the MNCC content continued to increase (Figure 11d).

**Figure 12:**
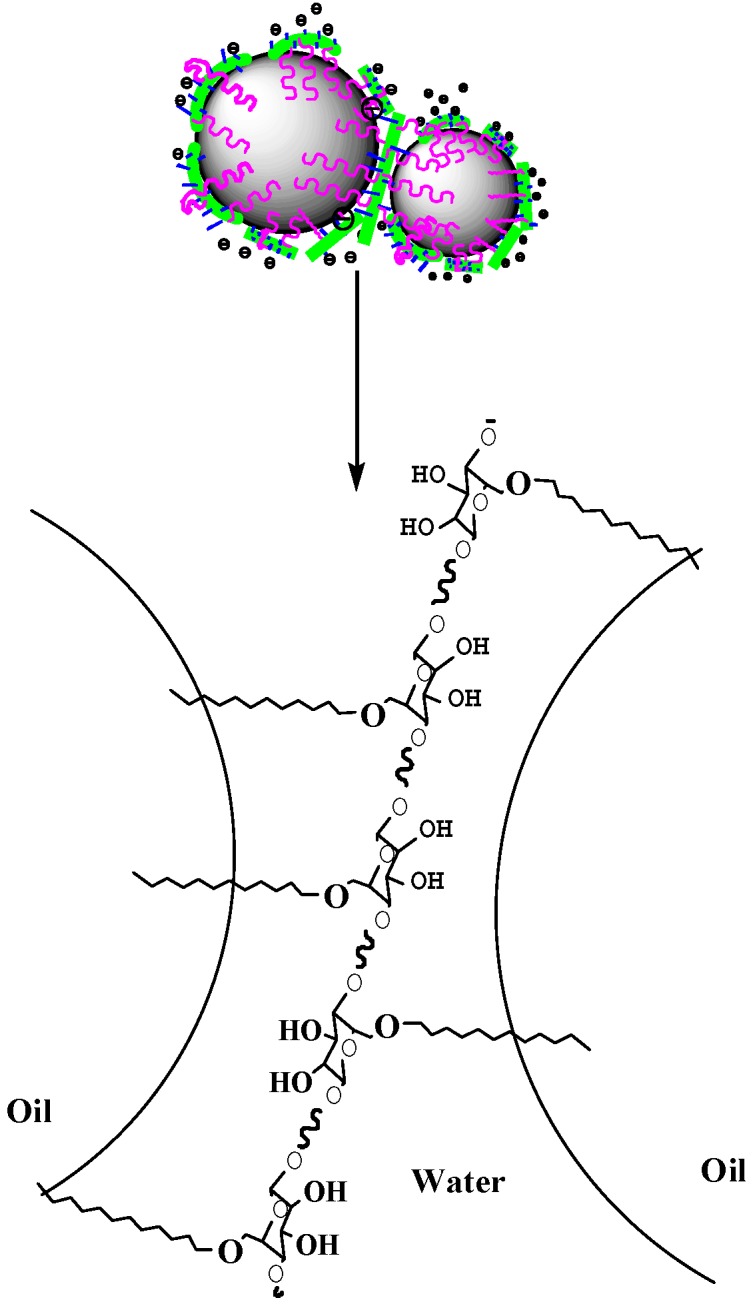
Schematic diagram of the adsorption of MNCC on styrene-acrylic emulsion oil-water interface.

Figure 10 showed the transmission electron microscope image, where styrene-acrylic emulsion droplets mostly existed in the form of two droplets stuck together. This phenomenon occurred because, when long-chain fatty acids were grafted to MNCC, they would be grafted on both sides of NCC. During the formation of the emulsion, the hydrophobic carbon long chain of the same MNCC macromolecule might be in the same oil phase droplet. It might also be adsorbed in two oil droplets, and the shape of two droplets stuck together was also formed, as shown in Figure 12. Due to the existence of double electric layers on the MNCC, the MNCC-coated styrene-acrylic emulsion droplets were formed, and, considering the electrostatic exclusion effect, coalescence did not occur easily. Hence, the styrene-acrylic emulsion droplets were stable in the water phase, and the styrene-acrylic emulsion exhibited a type of stability.

The stability of the emulsion was related to the following factors: the physical properties of the interfacial film, the electrostatic repulsion effect between the droplets, the space blocking effect of the polymer film, the viscosity of the continuous phase, the size and distribution of the droplet, and the volume ratio of the phase and the temperature. Among these factors, the physical properties, electrical properties, and space hindrance of the interfacial film were the most important. The surface MNCC showed negative electricity and had a certain zeta potential. The electrostatic repulsion between MNCC was conducive to the stability of the emulsion. As shown in Figure 11, MNCC was used as the interfacial layer between emulsion droplets and the aqueous phase, which forms a thick liquid affinity protective layer around the droplet. This protective layer constituted a space obstacle for the styrene-acrylic emulsion droplet to approach and contact. The hydrophilicity of the surface hydroxyl groups of the MNCC made the continuous phase liquid in the protective layer similar to that of the gel. The interface area formed a space barrier, which was conducive to the stability of the emulsion. Moreover, the MNCC had a certain size. The particle size of the MNCC was approximately 240 nm. The MNCC occupied a large area on the droplet surface, which was much larger than that of small molecular surfactants (0.27–0.45 nm^2^). Moreover, the particle size of styrene-acrylic emulsion stabilized by MNCC was larger than that of traditional surfactant stabilized styrene-acrylic emulsion. A big droplet relative to the small droplets, the interfacial area was smaller, the interfacial energy was lower, and the thermodynamic stability was greater.

Therefore, the stability of the styrene-acrylic emulsion was caused by electrostatic repulsion between MNCC, the hydrophilic lipophilic ability of MNCC, and the space barrier of the MNCC adsorption layer. Thus, MNCC could be applied to the stability of emulsions.

## 4. Conclusions

In this study, lauric acid, palmitic acid, and stearic acid were used to graft and modify NCC. The MNCC was used as a stabilizer to prepare styrene-butyl acrylate emulsion. The stability of emulsion was determined, and the mechanism of MNCC-stabilized emulsion was analyzed, according to the zeta potential and transmission electron microscope image. The following results were obtained. The average size of MNCC ranges from 244 to 255 nm. In the infrared images, ester bonds were formed, and XRD images showed slight changes compared with NCC. These findings indicate that long fatty acid chains were well grafted onto NCC through esterification initiated at low temperature. When the dosage of three MNCC was 0.05%, the stable styrene-acrylate emulsion was obtained with conversion rates of 92.5%, 94.2%, and 96.3%, respectively. The emulsion had good physical and chemical stability. The electrostatic exclusion of MNCC, the hydrophile lipophilic property of MNCC, and the spatial hindrance of the MNCC adsorption layer worked together to provide good stability for the styrene-acrylic emulsion, according to the measurement of the particle size, Zeta potential, and optical and transmission electron microscopies. In addition, MNCC can be applied to the stability of emulsions and could replace the application of surfactants, which provides an important theoretical basis for the application of MNCC in the field of food and medicine. It is also conducive to the conservation of fossil resources and the reduction of environmental pollution.

## Figures and Tables

**Table 1:** Proportion of emulsion polymerization reactants.

Monomers and Reactants	Ratio of the Reaction (wt%)	Mass of Reactants (g)
styrene	20	10
butyl acrylate	20	10
ammonium persulfate	1	0.5
MNCC	0–0.15	0–0.075
hydroquinone	0.001	0.0005
water	≈58.95	29.48

**Table 2:** Conditions and results of emulsion stability test.

**Ca^2+^ stability test (A)**	**Dosage of L-MNCC**	0.01%	0.025%	0.05%	0.075%	0.1%
	**Stability**	Layered, oil occupies 1/3	Layered, oil occupies 1/4	Not layered, stable	Not layered, stable	Not layered, stable
**pH stability test (B)**	**pH**	3	5	7	9 (NaOH)	11 (NaOH)
	**Stability**	Not layered, Stable	Not layered, stable	Not layered, stable	Layered, Slight flocculation	Layered, flocculated
**Dilution stability test (C)**	**Dilution multiple**	1000	100	50	10	5
	**Stability**	Not layered, stable	Not layered, stable	Not layered, stable	Not layered, stable	Not layered, stable
**Centrifugal oscillation stability (D)**	**Centrifugal rate**	5000 r/min	4000 r/min	3000 r/min	2000 r/min	1000 r/min
	**Stability**	Layered, oil occupies 1/4	Not layered, stable	Not layered, stable	Not layered, stable	Not layered, stable
